# Cross-Scale Modeling of CFRP Stacking Sequence in Filament-Wound Composite Pressure Vessels: In-Plane and Inter-Layer Homogenization Analysis

**DOI:** 10.3390/ma18194612

**Published:** 2025-10-05

**Authors:** Ziqi Wang, Ji Shi, Xiaodong Zhao, Hui Li, Huiming Shen, Jianguo Liang, Jun Feng

**Affiliations:** 1 National Key Laboratory of Transient Physics, Nanjing University of Science and Technology, Nanjing 210094, China; wzq190202@163.com (Z.W.); shiji15189805061@163.com (J.S.); 2 Institute of Industrial Science, The University of Tokyo, Tokyo 1538505, Japan; zhaoxiaodong0028@link.tyut.edu.cn; 3 College of Mechanical Engineering, Taiyuan University of Technology, Taiyuan 030024, China; lihui951107@163.com; 4 College of Nuclear Engineering, Rocket Force University of Engineering, Xi’an 710025, China; shenhuiming202@163.com

**Keywords:** carbon fiber-reinforced plastic, type IV composite pressure vessel, cross-scale modeling, FW cross-undulation RVE, inter-layer stacking sequence, burst pressure, fatigue life

## Abstract

Composite pressure vessels have attracted significant attention in recent years owing to their lightweight characteristics and superior mechanical performance. However, analyzing composite layers remains challenging due to complex filament-winding (FW) pattern structures and the associated high computational costs. This study introduces a homogenization method to achieve cross-scale modeling of carbon fiber-reinforced plastic (CFRP) layers, accounting for both lay-up sequence and in-plane FW diamond-shaped form. The stacking sequence in an FW Type IV composite pressure vessel is numerically investigated through ply modeling and cross-scale homogenization. The composite tank structure, featuring a polyamide PA66 liner, is designed for a working pressure of 70 MPa and comprises 12 helical winding layers and 17 hoop winding layers. An FW cross-undulation representative volume element (RVE) is developed based on actual in-plane mesostructures, suggesting an equivalent laminate RVE effective elastic modulus. Furthermore, six different lay-up sequences are numerically compared using ply models and fully and partially homogenized models. The structural displacements in both radial and axial directions are validated across all modeling approaches. The partial homogenization method successfully captures the detailed fiber-direction stress distribution in the innermost two hoop or helical layers. By applying the Hashin tensile failure criterion, the burst pressure of the composite tank is evaluated, revealing 7.56% deviation between the partial homogenization model and the ply model. Fatigue life analysis of the Type IV composite pressure vessel is conducted using ABAQUS^®^ coupled with FE-SAFE, incorporating an S-N curve for polyamide PA66. The results indicate that the fatigue cycles of the liner exhibit only 0.28% variation across different stacking sequences, demonstrating that homogenization has a negligible impact on liner lifecycle predictions. The proposed cross-scale modeling framework offers an effective approach for multiscale simulation of FW composite pressure vessels, balancing computational efficiency with accuracy.

## 1. Introduction

As a clean energy source, hydrogen burns to produce only water, with no pollution throughout the process [1]. Compared with fossil fuels, it not only has a higher energy density but also has an extremely wide range of sources—it can be obtained through various methods, such as fossil fuel reforming, industrial by-products, and water electrolysis [2]. Because of these advantages, the development of hydrogen energy has been receiving increasing attention worldwide [3]. Hydrogen storage vessels, as core equipment in the field of hydrogen energy, are a key link in supporting the large-scale application of the hydrogen energy industry chain and promoting the popularization of hydrogen storage and transportation [4,5]. Among hydrogen storage vessels, carbon fiber-reinforced plastic (CFRP) pressure vessels show significant advantages over traditional metal vessels: relying on high specific strength, they can achieve lightweight design under high-pressure hydrogen storage conditions, completely solving the problem of excessive weight of traditional metal vessels; at the same time, their excellent chemical inertness can effectively avoid hydrogen embrittlement, and they have a longer fatigue life, which significantly improves the economic efficiency while ensuring the safety of hydrogen storage [6]. However, carbon fiber pressure vessels currently have the problem of high price. To promote their large-scale application and ensure they are economical, reducing hydrogen storage costs has become a core task, which requires rational design of the structure of composite pressure vessels [7,8,9].

The composite pressure vessel is one of the most important parts in many kinds of hydrogen pressure vessels [10]. Composite overwrapped pressure vessels (COPVs) have a huge weight advantage over all-metal vessels, as they are only 50% of the weight of all-metal pressure vessels on the premise of meeting usage requirements [11]. The Type IV composite pressure vessel uses a polymeric liner fully wrapped with a fiber–resin composite for hydrogen transport and storage [12]. The liner of the composite pressure vessel acts as a leakproof measure, bearing little load. An important aspect of composite pressure vessels is the complexity of composite layer properties, which are determined by the anisotropic material properties of carbon fiber. The modeling and finite element analysis of the tank can be realized through specific software [13], but the analysis process is still complicated, and the model is different from the practical structure [14]. The design parameters of the composite layer affect the performance of the composite pressure vessel. The fiber volume fraction, layering sequence, winding angle, and fiber tension during filament winding are the factors [15,16,17,18] that must be considered in the design of the composite pressure vessel, which will affect the burst pressure. Carbon fiber is expensive and accounts for 50∼70% of the total cost of composite pressure vessels. Experiments require enormous material consumption, and numerical modeling is not always ideal [19]. Therefore, finding a method that can simplify the modeling of composite pressure vessels and accurately predict their properties is the key to solving this dilemma.

The homogenization method is widely used to simplify the analysis of composite structures based on rigorous mathematical derivation [20,21]. For the regions of interest, the most realistic modeling approach can be adopted, while for most of the other regions that are not of interest, the complexity and computational cost of the model can be greatly reduced by using homogenized material properties. The homogenization method can bridge the microscopic, mesoscopic, and macroscopic models and directly or indirectly study the mechanical properties [22,23,24] and damage mechanism [25,26,27] of composites through cross-scale analysis. The composite layers of pressure vessels are structurally similar to conventional laminates, which are stacked in a more complex way. Considering the homogenization method applied to the composite layer of the composite pressure vessel and studying the pressure performance of the model as a whole, the modeling of the tank can be simplified [28,29], and reliable analysis results can be obtained.

The structural design of composite pressure vessels is more challenging than that of metal pressure vessels. The properties of composites are inherited from their constituent materials and depend on filament-winding (FW) manufacturing processes, material properties, geometric configurations, etc., which leads to uncertainties at different scales. At present, many studies have been carried out to solve these design problems. Recently, Almeida et al. [30] used genetic algorithms to optimize the lay-up sequence of internally pressurized cylindrical shells to improve their strength. Nebe et al. [31] systematically investigated the influence of stacking sequence on the performance of composite pressure vessels, with particular focus on laminate quality, structural deformation, and burst pressure. In a complementary study, Hu et al. [16] conducted a comprehensive evaluation of how different stacking sequences affect the ultimate strength of composite vessels, employing a progressive damage model based on Hashin failure theory to accurately predict burst pressure. Further advancing this research area, Li et al. [32] examined the impact of various stacking sequences on the burst pressure of hydrogen storage vessels under fatigue loading conditions while also considering the effect of autofrettage pressure. Their study utilized a progressive damage model incorporating the Hashin failure criterion for burst pressure prediction, with experimental validation provided through hydraulic burst tests. Using the optimal stacking sequence is a key step in designing structures with the most appropriate mechanical properties.

Czapliński et al. [28] used a representative volume element (RVE) to predict the mechanical properties of unidirectional carbon fiber-reinforced plastic. Through the homogenization of the microscopic RVE, they derived the mechanical properties of the mesoscopic model and established a composite pressure vessel model. Qi et al. [33] predicted the mechanical properties of unidirectional CFRP using an RVE and further deduced the mechanical characteristics of laminates with different ply angles. They experimentally validated the rationality of predicting mesoscopic model mechanical properties from the microscopic RVE. Omairey et al. [34] developed an ABAQUS^®^ plug-in for RVE homogenization, making it more convenient to obtain the mechanical properties of mesoscopic models from microscopic homogenization. Lin et al. [35] employed a method to derive mesoscopic mechanical properties from microscopic homogenization and established a macroscopic model based on mesoscopic mechanical properties. They conducted a progressive failure analysis of composite pressure vessels, simulating the damage process of composite layers during hydrostatic burst tests. Rafiee et al. [36] established a macroscopic model through microscopic homogenization and then applied a downscaling approach, using a microscopic RVE to study the failure of macroscopic pressure vessels. Takemoto et al. [37] proposed a burst pressure prediction method for CFRP pressure vessel layers based on their mesostructure formed by filament winding, focusing on the microstructure of CFRP pressure vessels. Rouf et al. [38] effectively predicted the strain-rate-dependent response of mesoscopic models using a microscopic RVE.

**Figure 1:**
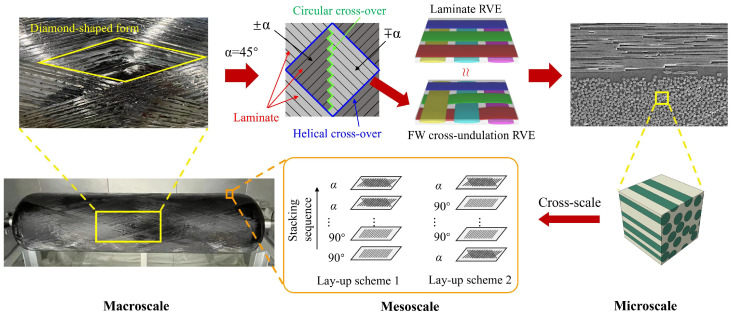
Cross-scale analysis of composite pressure vessel CFRP layers: in-plane RVE and lay-up homogenization.

In summary, scholars have made outstanding contributions to the homogenization-based analysis of composite layers of pressure vessels, and the stacking sequence of CFRP layers in composite pressure vessels has been extensively studied. Nevertheless, most studies only focus on one-stage homogenization, bridging microscale carbon fiber with composite-layer mesoscale modeling, and there still exist some points that require further discussion. Most numerical simulations consider the filament-wound (FW) composite layers as laminates for the sake of computational cost [39]. From a macroscale view, the typical RVE of in-plane CFRP mesostructure is characterized by a diamond-shaped form, where the winding angle is 45°, resulting in an FW cross-undulation RVE that has laminate (±α and ∓α), circular, and helical cross-over, as shown in Figure 1. The CFRP inter-layer structure can be considered as various lay-up schemes, which may affect the vessel’s structural performance. Therefore, it is interesting to use the homogenization method to check whether laminate modeling is reasonable to represent the actual in-plane FW cross-undulation RVE. Moreover, lay-up homogenization may also help to examine the stacking sequence effect on the mechanical properties of the composite pressure vessel. Aiming to address these problems, this paper adopts two-stage homogenization: first-stage homogenization bridges the mesoscale unit cells with the microscale fiber-matrix RVE in Figure 1, and second-stage homogenization applies to the in-plane FW CFRP, as well as inter-layer homogenization, which greatly simplifies the microstructure geometry and retains the key mechanical properties. Then the net theory design of a 70 MPa Type IV hydrogen storage pressure vessel is conducted, and ply and homogenized models for CFRP layers are validated in terms of strain–pressure response. The two-stage homogenization may shed some light on the stacking sequence effect on the pressure vessel’s mechanical performance, and liner fatigue life is further studied.

## 2. Analytical Homogenization

Carbon fiber-reinforced plastic exhibits inherent heterogeneity at the microscale, consisting of distinct fiber and matrix phases. To investigate its material properties, the representative volume element approach is commonly employed in micromechanical analyses. Classical micromechanics treats macroscopic material properties as homogeneous yet undetermined parameters, which are subsequently derived through homogenization techniques. These homogenized properties serve as critical input parameters for finite element simulations, enabling efficient numerical analysis of composite structures (Figure 1).

The homogenization approach offers significant advantages for composite material studies by explicitly accounting for microstructural characteristics. The development of homogenization theories has evolved considerably from the initial rule of mixtures, incorporating increasingly sophisticated microstructural representations. Among existing methodologies, asymptotic homogenization with thermal stress and the boundary force method [40] have emerged as predominant theoretical frameworks.

At the microscale, the composite is modeled as a periodic arrangement of uniform unit cells, while the macrostructure is conceptualized as a regular repetition of these fundamental units. Mathematical homogenization techniques enable the decoupling of linear elastostatic problems with periodic coefficients into separate fine-scale and coarse-scale formulations. This multiscale decomposition facilitates efficient computation of effective material properties while preserving essential microstructural information.

**Figure 2:**
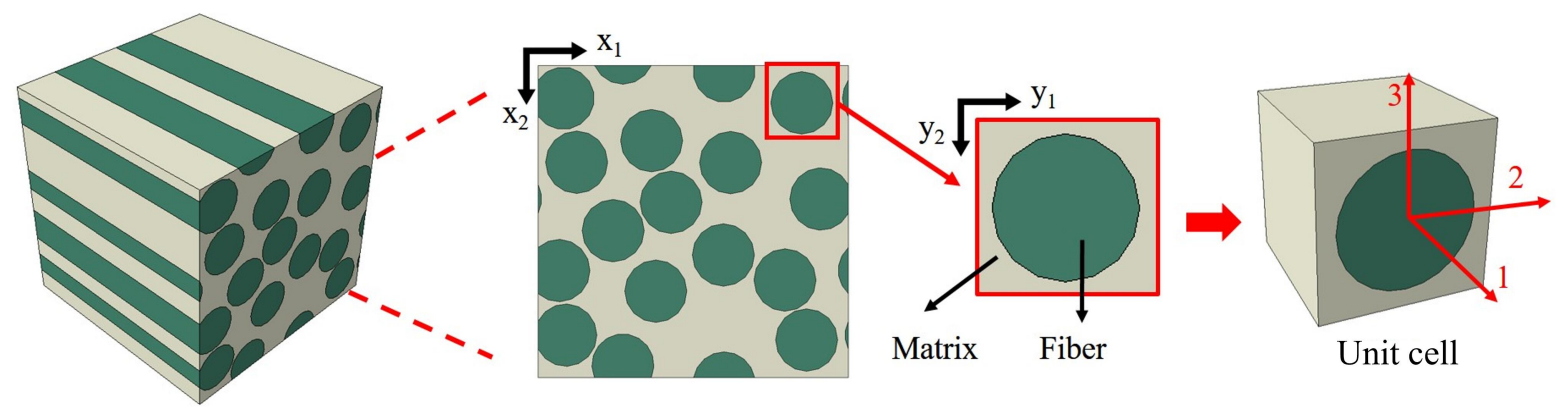
Unidirectional carbon fiber-reinforced plastic microstructure and unit cell with single fiber.

Composite structures are modeled as periodic arrangements of unit cells at the microscale. The position of any point is described by macroscopic coordinates $x=(x_{1},x_{2})$ and microscopic coordinates $y=(y_{1},y_{2})$, illustrated in Figure 2, with a 7 μm diameter for the carbon fiber. The scaling parameter ϵ links these coordinate systems.

(1) $$\epsilon =\frac{x}{y}$$

The displacement field *u* can be asymptotically expanded in terms of ϵ as

(2) $$u^{\epsilon }\left(x\right)=u\left(x,y\right)=u^{0}\left(x,y\right)+\epsilon u^{1}\left(x,y\right)+\epsilon^{2}u^{2}\left(x,y\right)+\cdots$$

The strain $\varepsilon_{ij}$ related to displacement *u* in Equation (2) can be derived using differentiation rules as follows:

(3) $$\begin{array}{cc} \varepsilon_{ij}\left(u^{\epsilon }\right) & =\frac{1}{2}\left(\frac{\partial u_{i}^{\epsilon }}{\partial x_{j}^{\epsilon }}+\frac{\partial u_{j}^{\epsilon }}{\partial x_{i}^{\epsilon }}\right)\\ \; & =\frac{1}{\epsilon }\varepsilon_{ij}^{\left(-1\right)}\left(x,y\right)+\varepsilon_{ij}^{\left(0\right)}\left(x,y\right)+\epsilon \varepsilon_{ij}^{\left(1\right)}\left(x,y\right)+\epsilon^{2}\cdots \end{array}$$

The material’s stiffness coefficient tensor is denoted as $C_{ijkl}^{\epsilon }$. In the constitutive model, the stress–strain relationship can be written as

(4) $$\sigma_{ij}^{\left(n\right)}\left(x,y\right)=C_{ijkl}^{\epsilon }\varepsilon_{ij}^{\left(n\right)}\left(x,y\right)\;\left(n=-1,0,1\right)$$

(5) $$\sigma_{ji}^{\epsilon }=C_{ijkl}^{\epsilon }\varepsilon_{kl}=\frac{1}{\epsilon }\sigma_{ij}^{\left(-1\right)}\left(x,y\right)+\sigma_{ij}^{\left(0\right)}\left(x,y\right)+\epsilon \sigma_{ij}^{\left(1\right)}\left(x,y\right)+\cdots$$

The fundamental equation in linear elasticity is the equilibrium equation under body force $f_{i}$ in domain Ω, which can be expressed as

(6) $$\sigma_{ij,j}+f_{i}=0\;in\;\Omega$$

To avoid repetition of different $u_{i}^{\left(0\right)}$ in finite element analysis, the characteristic function $\left(\chi_{k}^{mn}\left(y_{i}\right)\right)$ is introduced to couple the macro displacement with the first-order displacement.

(7) $$u_{i}^{\left(1\right)}=\chi_{i}^{kl}\frac{\partial u_{k}^{\left(0\right)}}{\partial x_{l}}$$

Considering that $u_{i}^{\left(n\right)}$ is a periodic function of the domain *Y*, the homogeneous stiffness coefficient $C_{ijmn}^{H}$ is defined as

(8) $$C_{ijmn}^{H}=\frac{1}{\left|Y\right|}\int_{Y}C_{ijkl}\left(\delta_{km}\delta_{ln}+\frac{\partial \chi_{k}^{mn}}{\partial y_{l}}\right)dY$$

Considering the symmetry of kl and the quantified mn, the following relation can be obtained:

(9) $$\frac{\partial }{\partial y_{j}}\left[C_{ijkl}\frac{1}{2}\left(\frac{\partial \chi_{k}^{mn}}{\partial y_{l}}+\frac{\partial \chi_{l}^{mn}}{\partial y_{k}}\right)\right]=-\frac{\partial C_{ijmn}}{\partial y_{j}}$$

To implement the boundary force method, multiply both sides of Equation (9) by $\partial \chi_{i}^{mn}$ and integrate over the unit cell domain, reformulating it into the governing equation form.

(10) $$\int_{Y}\frac{\partial }{\partial y_{j}}\left[C_{ijkl}\frac{1}{2}\left(\frac{\partial \chi_{k}^{mn}}{\partial y_{l}}+\frac{\partial \chi_{l}^{mn}}{\partial y_{k}}\right)+\frac{\partial C_{ijmn}}{\partial y_{j}}\right]\delta \chi_{i}^{mn}dV=0$$

An alternative formulation of the homogenization coefficient can be derived by applying Gauss’s theorem.

(11) $$C_{ijmn}^{H}=\frac{1}{\left|Y\right|}\int_{Y}C_{ijkl}\frac{1}{2}\left(\frac{\partial \chi_{k}^{mn}}{\partial y_{l}}+\frac{\partial \chi_{l}^{mn}}{\partial y_{k}}\right)dV+\frac{1}{Y}\int_{Y}C_{ijmn}dV$$

**Figure 3:**
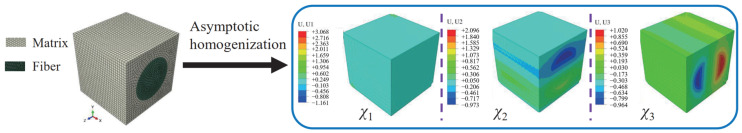
Displacement field of unit cell under 3 different thermal loads.

In Equation (11), the homogenization coefficient in the boundary force method is obtained by summing the volume-averaged stress and the volume-averaged elastic tensor within a unit cell. For CFRP materials consisting of carbon fibers and matrix, this coefficient includes two components: (1) the average elastic modulus of the structure and (2) the average additional stress induced by material heterogeneity in the unit cell. The asymptotic homogenization process can be efficiently implemented using commercial finite element software, as illustrated in Figure 3, where the characteristic function is the result of the displacement field for Equation (11) calculation.

## 3. Geometric Design of Composite Pressure Vessel

### 3.1. Winding Angle and Thickness

The liner of the Type IV composite pressure vessel is made of polyamide, and the outer liner is wrapped with multilevel CFRP. The filament-wound carbon fiber bundle in the region of the dome with complex geometry is characterized by various angles and thickness. Generally, netting theory is adopted to design composite layers of pressure vessels, where the inner pressure is borne almost entirely by CFRP layers. In netting theory, it is assumed that the fibers are uniformly distributed and the fiber direction forces are the same. It is convenient to accurately calculate the CFRP thickness beyond two band widths at the dome. However, due to slip, porosity, and bonding, the predicted value deviates greatly from the actual value near the polar hole. In order to verify the simplified model by the homogenization method, it is necessary to conduct ply modeling and analysis of the composite pressure vessel. In this study, the cubic spline function method and the double formula method are combined to forecast the CFRP thickness within two bandwidths and beyond two bandwidths, respectively.

Within two bandwidths, the region of the polar hole theoretically has a large accumulation of fibers, while in practice, the dome curve is smooth due to fiber slip and bridging. The method of spline interpolation has proven to be reasonable by practical measurement and investigations in the literature. Suppose that the cubic spline function $t(r_{i})$ in the radius interval $\left[r_{0},R\right]$ is

(12) $$t\left(r_{i}\right)=m_{1}\times r_{i}^{0}+m_{2}\times r_{i}^{1}+m_{3}\times r_{i}^{2}+m_{4}\times r_{i}^{3}$$

where $m_{i}\left(i=1,2,3,4\right)$ are undetermined coefficients; *r* is the radius of the latitude circle near the polar hole. Four boundary conditions are found to solve for undetermined coefficients:

Condition 1: The number of fiber bundles at the pole hole is equal to the number in the cylinder due to the continuity of the filament-winding process. According to the two bundles of the cylinder corresponding to a bundle near the pole hole, the following can be obtained:

(13) $$t\left(r_{0}\right)=t_{R}\cdot m_{R}/\left(2\times m_{0}\right)=\sum_{i=1}^{4}m_{i}\times r_{0}^{i-1}$$

where $r_{0}$ is the radius of the polar hole; $t_{R}$ is the winding thickness of the cylinder; $m_{R}$ is the number of bundles in each layer of the cylinder; and $n_{0}$ is the number of bundles near the pole hole.

Condition 2: Fiber thickness at two bandwidths can be predicted by the double-formula method,

(14) $$\begin{array}{c} t\left(r_{2b}\right)=\frac{arccos\left(r_{0}/r_{2b}\right)-arccos\left(r_{b}/r_{2b}\right)}{arcsin[\frac{\sqrt{{\left(\sqrt{R^{2}-r_{0}^{2}}-\sqrt{R^{2}-r_{b}^{2}}\right)}^{2}+b^{2}}}{2R}]}\times t_{R}=\sum_{i=1}^{4}m_{i}\times r_{2b}^{i-1} \end{array}$$

where $r_{b}$ is the radius of the latitude circle at a bandwidth; $r_{2b}$ is the radius of the latitude circle at two bandwidths; *R* is the radius of the cylinder; and *b* is the width of the bundle.

Condition 3: Since the fiber-winding molding at the dome is smooth and continuous in practice, the function should be continuously differentiable.

(15) $$\begin{array}{c} \frac{dt(r_{2b})}{dt}=\frac{\left(\frac{r_{0}}{r_{2b}\times \sqrt{r_{2b}^{2}-r_{0}^{2}}}-\frac{r_{b}}{r_{2b}\times \sqrt{r_{2b}^{2}-r_{0}^{2}}}\right)\times t_{R}}{arcsin[\frac{\sqrt{{\left(\sqrt{R^{2}-r_{0}^{2}}-\sqrt{R^{2}-r_{b}^{2}}\right)}^{2}+b^{2}}}{2R}]}=m_{2}+2m_{3}r_{2b}+3m_{4}r_{2b}^{2} \end{array}$$

Condition 4: The number and volume of the bundle remain constant during the winding process.

(16) $$\begin{array}{c} \int_{r_{0}}^{r_{2b}}2\pi rt\left(r\right)dr=\int_{r_{0}}^{r_{2b}}2\pi r\cdot \left(m_{1}\times r^{0}+m_{2}\times r^{1}+m_{3}\times r^{2}+m_{4}\times r^{3}\right)dr \end{array}$$

Combining the thickness formula in the double-formula method and the above four boundary conditions, the fiber thickness near the pole hole (usually two bandwidths) can be predicted. The fiber thickness outside two bandwidths is predicted at the dome based on the geometric method.

(17) $$t\left(r\right)=\frac{m_{R}\cdot n_{R}}{\pi }\cdot \left[arccos\left(\frac{r_{0}}{r}\right)-arccos\left(\frac{r_{0}+b}{r}\right)\right]\cdot t_{P}$$

where $t_{P}$ is the thickness of a single yarn. The fiber accumulation thickness distribution of the dome can be obtained by combining the above theories. Note that there are deviations between theory and practice, and smoothing by the equal-volume method is still necessary for modeling.

**Figure 4:**
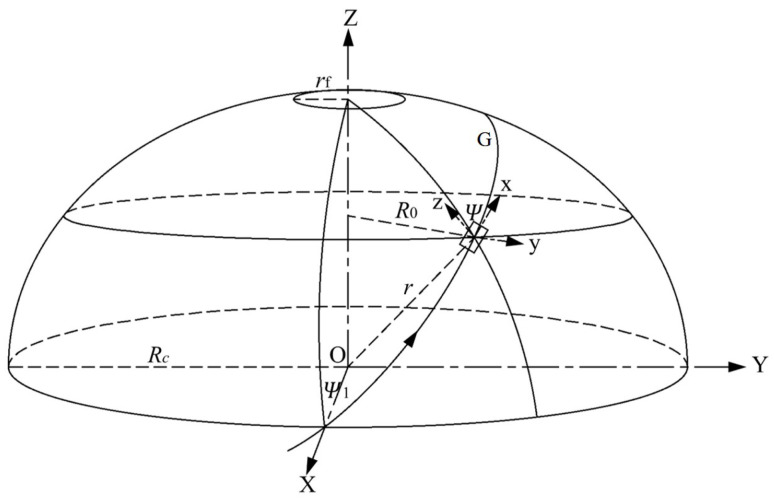
A schematic diagram of the geodesic on the surface of a rotating body.

In geodesic theory, suppose G is the geodesic on the surface of the body of revolution, as shown in Figure 4. The spatial Cartesian coordinate systems O(x,y,z) and O(X,Y,Z) are established. The former is a static coordinate system and the latter is a dynamic coordinate system. On the surface of the body of revolution, the geodesic curve can be determined by the Clairaut relation [41].

(18) $$R_{0}\cdot sin\left(\psi \right)=R_{c}\cdot sin\left(\psi_{1}\right)=constant$$

The filament-winding angle at the dome is 90°, that is, ψ= 90°, so

(19) $$R_{0}\cdot sin\left(\psi \right)=r_{f}=R_{c}\cdot sin\left(\psi_{1}\right)$$

where $\psi_{1}$ is the winding angle of the cylinder, ψ is the winding angle of the dome, and $r_{f}$ is the radius of the polar hole.

For the composite pressure vessel with the same pole hole size $r_{0}$ at both ends, the winding angle α of arbitrary radius *r* during geodesic winding is

(20) $$sin\alpha =\frac{r_{0}}{r}$$

The composite layer of the pressure vessel cylinder is regarded as a thin-walled tube, and the theoretical fiber-winding thickness obtained by membrane netting theory is

(21) $$t_{\alpha }=\frac{RP_{max}}{2\left[\sigma \right]kcos^{2}\alpha_{1}}$$

(22) $$t_{\theta }=\frac{RP_{max}}{2[\sigma ]}\left(2-tan^{2}\alpha_{1}\right)$$

where $t_{\alpha }$ is the thickness of the helical winding; $t_{\theta }$ is the thickness of the hoop winding; *R* is the radius of the cylinder; $P_{max}$ is the burst pressure of the composite pressure vessel; [σ] is the allowable stress of the composite; $\alpha_{1}$ is the winding angle of the cylinder; and *k* is the introduced enhancement coefficient. The strength of the actual composite layer cannot reach the ideal state due to the influence of the winding process, path deviation, fiber crossing, and other factors, taking k=0.8 based on experience.

### 3.2. Type IV Pressure Vessel Design

**Figure 5:**
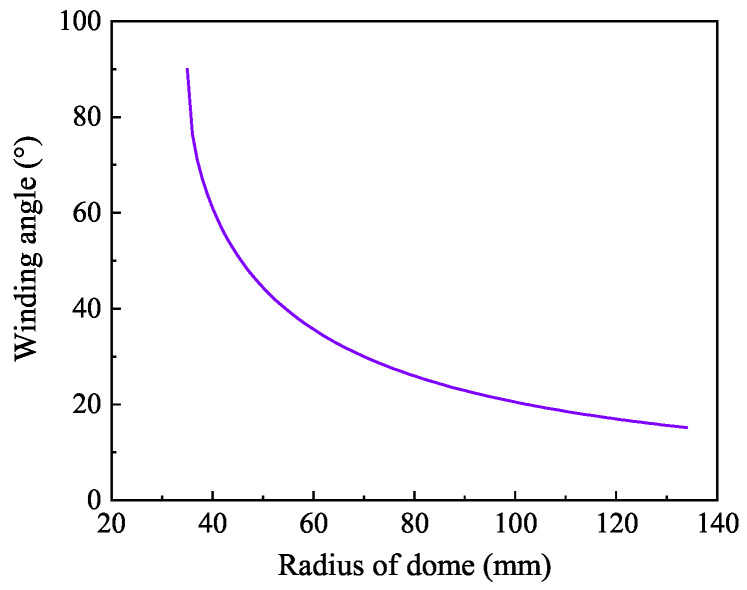
The theoretical variation in the winding angle of the dome with the radius of the latitude circle.

Not only are material properties complex in the design of composite layers, but the winding parameters are also variable. Helical winding and hoop winding can be realized on the tank cylinder, but hoop winding cannot be realized in the region of the dome. In this paper, the carbon fiber is T700SC-12K while the resin is 914 epoxy, and the producer-provided material parameters for fibers and the matrix are listed in Table 1. While the fiber volume fraction is 50%, the first-stage homogenized material properties in Table 2 at the micro level satisfy the requirement for input data, which can be applied to the meso-level laminate. By using the above theories with some initial data, shown in Table 3, the fibers’ distribution pattern at the dome can be obtained. The particularity of the dome structure determines the modeling complexity; the angle varies with the latitude circle’s radius, as shown in Figure 5, and the allowable pressure of the tank is 70 MPa. The change in winding angle near the pole hole is sharp, while that near the cylinder is gentle. The dome is divided into 14 regions according to the radius of the latitude circle, and the winding angle of each region is assumed to be constant. The purpose of segmentation is to facilitate modeling and to ensure reliability.

**Figure 6:**
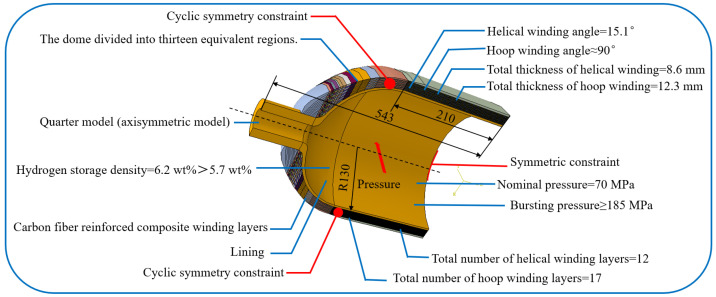
Selection and establishment of model.

The tank, as an axisymmetric model, can be constructed as only one quarter of the total model. Cyclic symmetric constraints and symmetric constraints are applied to the model to ensure a reasonable analysis process, as shown in Figure 6. To meet the requirement of 70 MPa nominal pressure and 185 MPa bursting pressure, the 260 mm inner diameter requires a helical winding angle $\alpha_{1}$ of 15.1°, 8.6 mm helical winding thickness, and 12.3 mm hoop winding thickness. Since the single-yarn thickness $t_{p}$ is 0.76 mm, the helical and hoop layer numbers are 12 and 17.

### 3.3. Validation of Pressure Vessel Design

**Figure 7:**
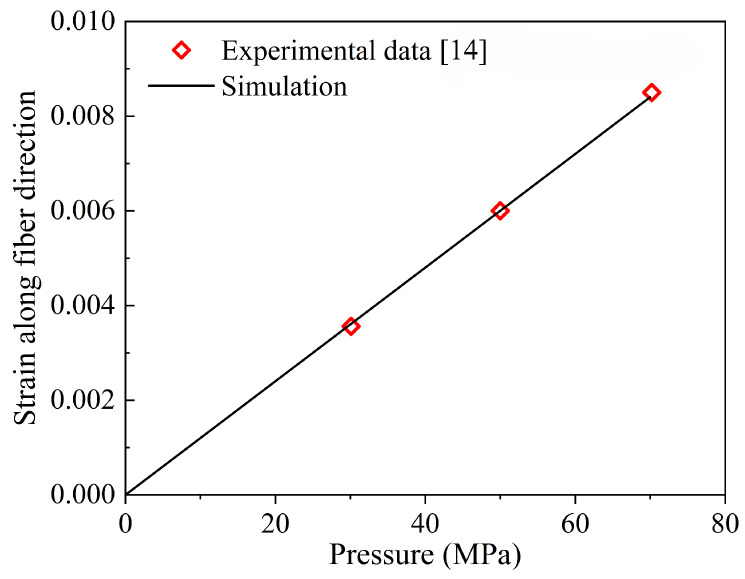
Comparison between theory and experimental strain responses of composite pressure vessel [14].

The element type of the tank model is C3D6, and the total number of elements is 122,360. This composite pressure vessel model is developed and meshed to study the mechanical behavior of the composite pressure vessel, which is the basis for evaluating the effectiveness of homogenization. In the elastic stage, the mechanical behavior of CFRP is similar, so the mesoscopic model of the composite pressure vessel is compared with the literature [14] to ensure the accuracy of the model. In order to verify it, the mode of alternate winding is determined, which is similar to that in the literature [14]. The foregoing hypothesis is validated in Figure 7, where the tested fiber-direction strain at different pressures agrees well with simulation results.

### 3.4. Lay-Up Sequences

**Figure 8:**
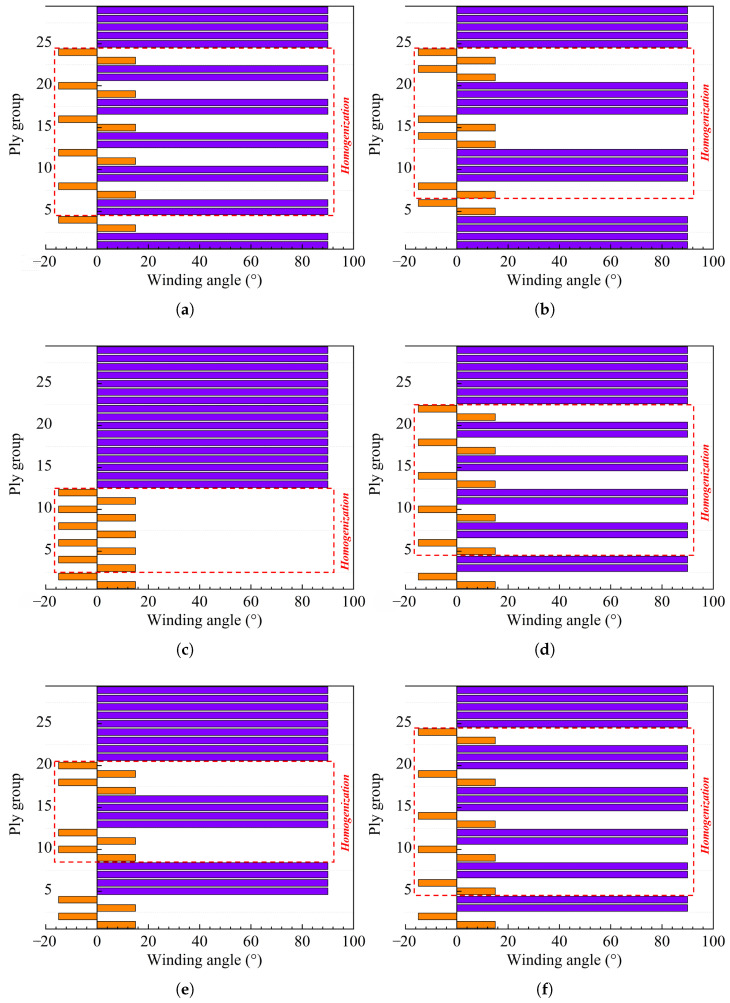
Lay-up design schemes. The orange color represents the hoop winding layer, and the purple color represents the helical winding layer. (**a**) Stacking sequence a, (**b**) stacking sequence b, (**c**) stacking sequence c, (**d**) stacking sequence d, (**e**) stacking sequence e, and (**f**) stacking sequence f.

To assess how different stacking sequences affect the strength of composite pressure vessels, a comparative study is conducted using multiple lay-up configurations. All design variants maintain identical quantities of hoop and helical layers, along with consistent composite thicknesses, with the sole variation being the layer stacking order. This approach aims to systematically investigate how each configuration influences the vessel’s structural performance. For clarity, the various stacking schemes are presented in a bar chart format (Figure 8), where helical layers alternate between positive and negative winding angles. In this work, six alternate stacking sequences are designed, namely, stacking sequence a ([${\left(90{}^{\circ}\right)}_{2}$, ±15.1°]_6_, ${\left(90{}^{\circ}\right)}_{5}$), stacking sequence b ([${\left(90{}^{\circ}\right)}_{4}$, ${\left(\pm 15.1{}^{\circ}\right)}_{2}$]_3_, ${\left(90{}^{\circ}\right)}_{5}$), stacking sequence c (${\left(\pm 15.1{}^{\circ}\right)}_{6}$, ${\left(90{}^{\circ}\right)}_{17}$), stacking sequence d ([${\left(15.1{}^{\circ}\right)}_{2}$, ${\left(\pm 90{}^{\circ}\right)}_{2}$]_6_, ${\left(90{}^{\circ}\right)}_{5}$), stacking sequence e ([${\left(15.1{}^{\circ}\right)}_{4}$, ${\left(\pm 90{}^{\circ}\right)}_{4}$]_3_, ${\left(90{}^{\circ}\right)}_{5}$), and stacking sequence f ([${\left(15.1{}^{\circ}\right)}_{2}$, ${\left(\pm 90{}^{\circ}\right)}_{2}$]_3_, [${\left(15.1{}^{\circ}\right)}_{2}$, ${\left(\pm 90{}^{\circ}\right)}_{3}$]_3_, ${\left(90{}^{\circ}\right)}_{2}$).

In Figure 8, six distinct stacking configurations are designed with different layer arrangements. Stacking sequences a and b feature a hoop layer as the innermost layer, while stacking sequences b, c, d, and e employ a helical layer as their innermost layer. A comparison between stacking sequences a and b, as well as between d and e, is conducted to evaluate the effect of simultaneously increasing the number of alternating hoop and helical layers on structural strength. Additionally, a comparison between stacking sequences d and f is performed to investigate the influence of solely increasing the number of hoop layers in the alternating winding sequence on the pressure vessel’s strength. Although the winding configuration of stacking sequence c will not be practically implemented in real-world applications, it is still included in the study for comparative analysis with the other schemes. For different stacking sequences, different inter-layer homogenization treatments, denoted by the red-dashed box in Figure 8, are used to improve the accuracy of homogenization.

**Figure 9:**
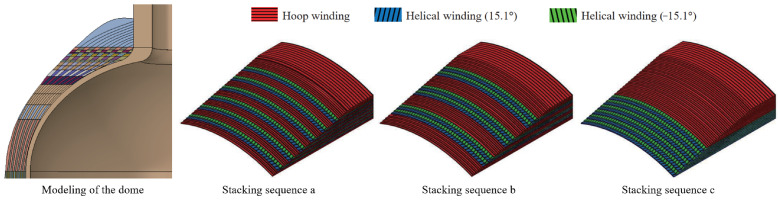
The processing of the dome and three different ways of winding the cylinder.

Figure 9 shows the modeling results of the dome and stacking sequences a, b, and c. There is no doubt that the modeling of composite pressure vessels is complex. A layer of composite material is very thin, and the composite layers have multiple directional characteristics, while the tank generally requires a large number of composite layers. The output of professional fiber-winding software is not exactly the same as reality [14]. In the case of predicting burst pressure, microscale information is often not required. Under the action of internal pressure, the stiffness response of composite layers is determined, which provides a basis for the application of the homogenization method. Modeling can be simplified at the macro level by using homogenization theory.

## 4. Cross-Scale Modeling of Pressure Vessel Composite Layer

The second-stage homogenization method applies the effective material parameters calculated for mesoscopic cell elements to the macroscopic model, avoiding the need to model the entire complex microstructure and significantly reducing computational cost, which indicates that the material properties of the RVE are the basis of composite modeling. The homogenized effective elastic properties of the RVE are determined via the EasyPBC plug-in in ABAQUS^®^ by applying suitable periodic boundary conditions.

**Figure 10:**
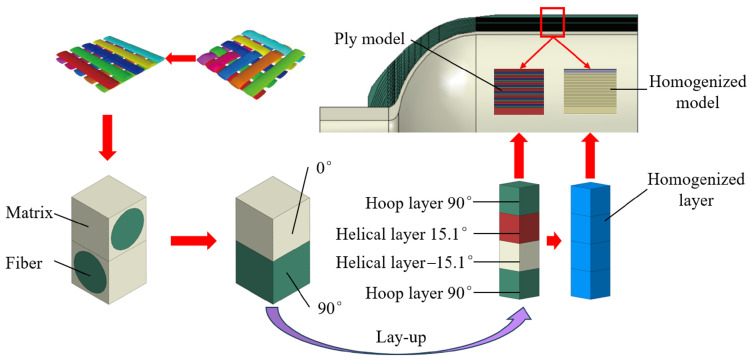
Actual in-plane and inter-layer structures and homogenization of FW composite layer.

For the composite winding layers, the fiber bundle in-plane structure at the mesoscopic level resembles a cross-undulation RVE, as illustrated in Figure 10. However, the approximated laminate model may differ from this actual in-plane FW cross-undulation structure, necessitating further validation of the reliability of the widely used ply modeling for the FW composite layer. Moreover, the inter-layer structure may be regarded as laminate with various orientations, which can also be treated as a ply model and a homogenized model, as shown in Figure 10.

### 4.1. In-Plane RVE Homogenization

Morozov’s articles report extensive work on the effects of mosaic filament-winding patterns on the composite structure strength [42,43], and indeed, the winding pattern exerts a significant effect on the mechanical response of filament-winding structures [44]. In this work, we focus on in-plane FW cross-undulation structures to study the homogenization effect.

**Figure 11:**
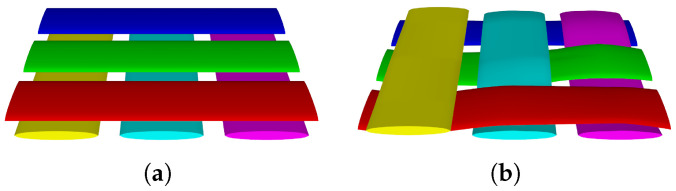
RVEs of laminate and in-plane FW cross-undulation structures. (**a**) ${\mathrm{RVE}}_{a}$, (**b**) ${\mathrm{RVE}}_{b}$.

**Figure 12:**
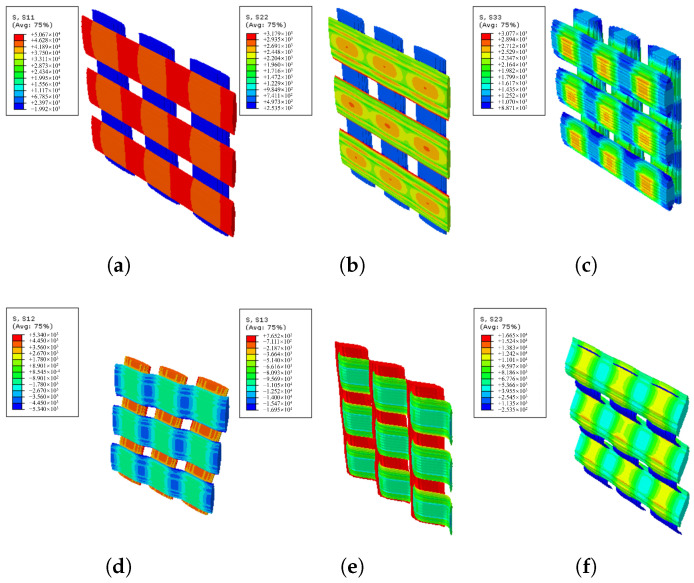
Stress field of laminate in-plane ${\mathrm{RVE}}_{a}$. (**a**) S11, (**b**) S22, (**c**) S33, (**d**) S12 and (**e**) S13, and (**f**) S23.

**Figure 13:**
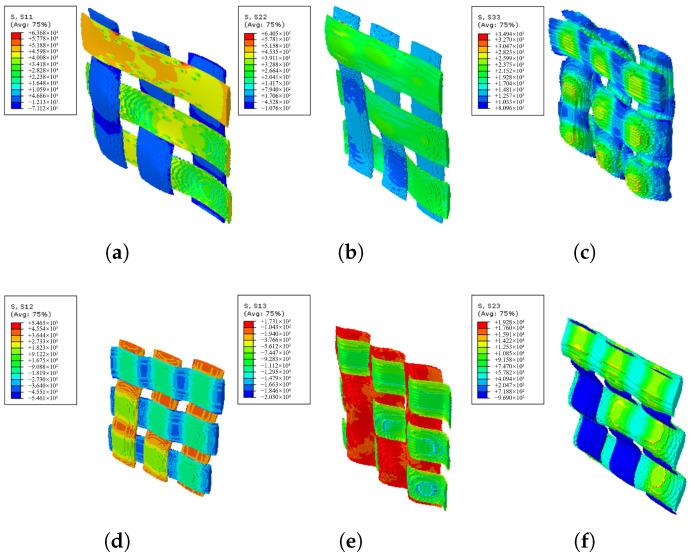
Stress field of in-plane FW ${\mathrm{RVE}}_{b}$. (**a**) S11, (**b**) S22, (**c**) S33, (**d**) S12 and (**e**) S13, and (**f**) S23.

Two kinds of RVEs, designated ${\mathrm{RVE}}_{a}$, ${\mathrm{RVE}}_{b}$, are developed to model the typical parts of the actual FW cross-undulation structure, as shown in Figure 11, where the solid part represents the carbon fiber yarns and the transparent part represents the matrix. The volume fraction and material parameters of two RVE models are consistent, and periodic boundary conditions are applied to both models. The stress distributions of ${\mathrm{RVE}}_{a}$ and ${\mathrm{RVE}}_{b}$ in directions 11, 22, 33, 12, 13, and 23 are illustrated in Figure 12 and Figure 13, with half of the model rendered as solid and the other half as transparent to enhance the visualization of stress distribution in fiber bundles. ${\mathrm{RVE}}_{a}$ is the orthogonal laminate, while ${\mathrm{RVE}}_{b}$ is the cross-undulation. Due to the zig-zag fiber geometry, the stress field of the FW cross-undulation RVE exhibits stress concentration, suggesting a stiffer equivalent modulus, especially in shear response. Notably, manufacturing-induced defects in filament winding as well as interface cohesion are also important for the composite vessel strength analysis, requiring further study for their consideration in RVE development.

The results for the homogenization parameters for the laminate and FW cross-undulation RVE models are presented in Table 4. Disregarding modeling errors, the material parameters obtained from laminate and FW cross-undulation RVE models are similar, which suggests that the effective material parameters of the unit cell are primarily controlled by the fiber volume content, with the differences between laminate and cross-undulation structures being negligible in terms of effective material parameters, allowing the use of a simple model instead of more complex in-plane winding structures for effective modeling. Additionally, at the macroscale, the winding layers of the pressure vessel can be replaced with simple laminar structures to simplify the model. After homogenization, the Young’s modulus of the FW cross-undulation RVE is 95.7% of that of the laminate RVE, implying that the zig-zag geometry may exert about a 4% effect on fiber-direction stress.

### 4.2. Inter-Layer Homogenization

**Figure 14:**
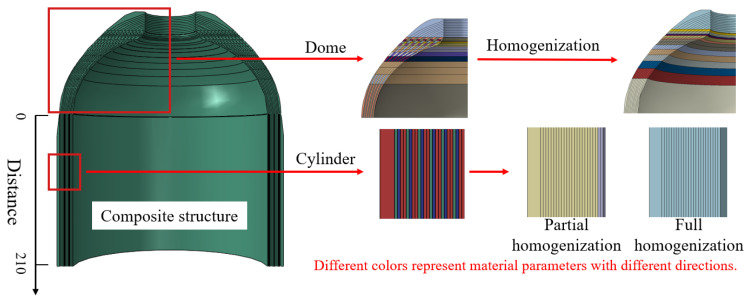
Homogenization treatment for dome and cylinder.

In the process of homogenization, the working mode of the composite pressure vessel cannot be ignored. Complex damage patterns influence burst pressure and burst mode, which are important aspects of the homogenized tank that need to be considered. In general, the composite layer accounts for almost all the load, and the advantage of using a plastic liner is that it reduces the weight of the tank. Fiber fracture is the most common failure mode and is accompanied by matrix cracking or delamination. Concerning burst modes, the damage starts at the innermost layer of the winding layers, which spreads gradually as the internal pressure increases and widespread failure occurs in a very short time. Therefore, the failure of the innermost composite layer is an important basis for judging the failure of the tank. This indicates that the failure of the composite layer does not occur simultaneously, but the damage extends from the inner layer to the outer layer. The partial homogenization method takes this damage form into account; it retains two hoop layers close to the liner, and the rest is homogenized, as shown in Figure 14. The inner layer of the composite is shown on the far left.

The tank can be divided into liner and composite material layers according to the structure; the liner structure is relatively simple and does not need to be homogenized. Due to the different structures of the dome and cylinder, different homogenization treatments are needed. For the dome region, the radius of the latitude circle within a certain range is treated as a whole for homogenization. The purpose of zonal processing is to maximize the consistency between the homogenized results and the initial state. Table 5 shows the homogenized moduli of different regions of the dome. Direction 1 is the reference direction (axial), and the corresponding modulus ($E_{11}$) decreases with the increase in the winding angle, while the transverse modulus ($E_{33}$) increases gradually. The variation in the homogenized modulus is closely related to fiber orientation.

For the cylinder, an important aspect of homogenization is how to deal with the ply model accurately. Considering the failure mode of the composite layer, the innermost layer usually fails first, followed by the failure of the rest of the composite layers. Full homogenization means that all composite layers have been evaluated for homogenized effective elastic moduli, and partial homogenization retains two hoop winding layers close to the liner, while the remaining layers are homogenized, as shown in Figure 14. Traditional full homogenization changes the innermost material properties, which may lead to unreliable results. So homogenization analyses are carried out, including full homogenization and partial homogenization of composite layers. Theoretically, full homogenization is less effective than partial homogenization.

**Figure 15:**
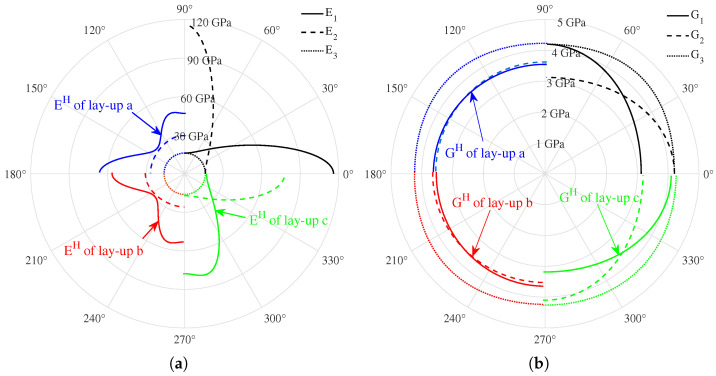
Moduli of CFRP inter-layer homogenization. (**a**) Young’s modulus of homogenization, (**b**) Shear modulus of homogenization.

For the safe failure mode, the failure of the composite pressure vessel occurs in the cylinder, and the inner helical winding layer first fails and then expands outward layer by layer until the tank breaks. This means that the homogenization of the composite layer is selective rather than blind. This work adopted the partial homogenization of the composite layer; i.e., the two innermost layers are modeled as mesoscale plies, while the alternating hoop and helical winding layers are modeled as homogenized composite, as illustrated in Figure 8, where the red-dashed lines denote the inter-layer homogenization regions. Stacking sequences a, b, and c after inter-layer homogenization are comparatively plotted in Figure 15, where $E^{H}$ and $G^{H}$ correspond to the homogenized layer in Figure 10. The inter-layer homogenization changes the in-plane mechanical properties $E_{1}^{H}$, $E_{2}^{H}$, $G_{1}^{H}$, and $G_{2}^{H}$ but leaves the thickness direction moduli $E_{3}^{H}$ and $G_{3}^{H}$ constant.

## 5. Cross-Scale Analysis of Lay-Up Sequence

### 5.1. Comparison of Mechanical Responses

**Figure 16:**
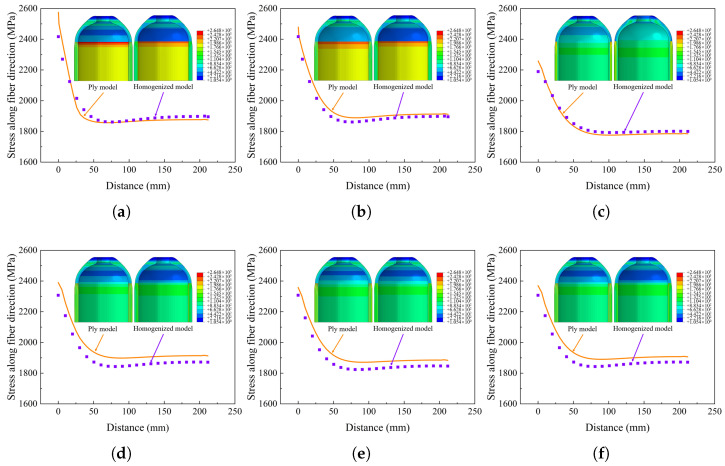
Comparison of stress in the cylinder: (**a**–**f**) stacking sequences a–f.

The stress value and distribution of the tank directly affect the performance of the structure, which is an important standard for verifying the homogenization method. Figure 16 illustrates the stress along the fiber direction of the whole composite structure. The stress along the fiber direction and distributions of stacking sequence a and stacking sequence b are similar, indicating that the blasting modes under the two stacking sequences are also similar. Note that this does not indicate that the burst pressure is the same, as different alternate winding sequences may produce different voids and stress concentrations, depending on the process. For stacking sequence c, helical winding followed by hoop winding is an extreme winding mode, and the stress distribution of the model treated with the partial homogenization method is in good agreement with the ply model. For homogenized models, full homogenization shows a smaller value of the first principal stress, which is undoubtedly less accurate compared with the ply models, because the stress in the innermost layer is averaged over other regions of the composite. Partial homogenization retains the structural characteristics of the innermost layer, while the influence of homogenization in other regions can be ignored, so the analysis result is accurate. The maximum stress occurs in the transition section between the dome and cylinder, so the first failure occurs in this region.

The analysis result shows that the stresses of different layering modes are different. The dome region can only realize helical winding and cannot realize hoop winding. So the winding results of the dome under different stacking sequences are similar. Figure 16 shows the stress along the fiber direction of the cylinder. The distance represents the axial direction away from the dome and cylinder transition region, as shown in Figure 14. For different stacking sequences, the stress distribution trend in the cylinder is similar, and the maximum stress along the fiber direction appears in the transition region. The stress distribution of the homogenized model is consistent with the ply model when using the partial homogenization method. It can be seen that the stress values of stacking sequence a, stacking sequence b, and stacking sequence c are more consistent with the ply model. At the same time, it shows that the homogenization of the alternating mode of hoop winding followed by helical winding is more accurate.

**Figure 17:**
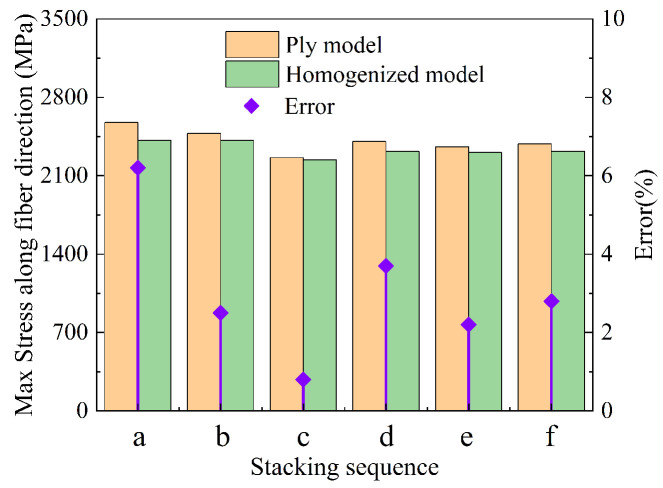
Max stress along the fiber direction and error. Homogenized model represents the partially homogenized model, and the horizontal axis represents the different stacking sequences, e.g., a represents stacking sequence a.

**Figure 18:**
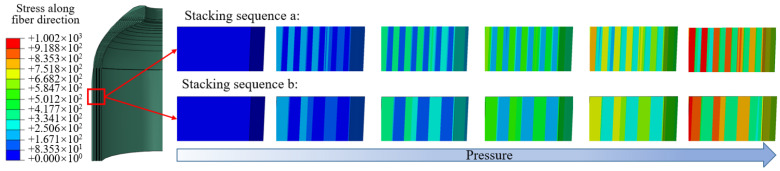
The change in stress along the fiber direction of the composite layers under a loading of 70 MPa with different alternate stacking sequences.

In general, the maximum stress along the fiber direction determines the bearing capacity of the tank, and the larger the stress is, the more likely it is that the composite layer will fail [45]. The mechanical response may also be controlled by the matrix in the circumferential direction [42,46]; fiber-direction stress is the response of concern for the composite structure [47]. For different stacking sequences, the partial homogenization method is adopted to simplify modeling and analyze the maximum stress along the fiber direction. As shown in Figure 17, it is found that the maximum stresses along the fiber direction are different among the different stacking sequences at 185 MPa pressure. The maximum stress is 2576 MPa and the minimum stress is 2262 MPa under the six stacking sequences. The stress of stacking sequence c is minimal because the hoop winding layer is distributed in the outermost ring, and part of the stress is shared by the helical winding layer near the liner, and the tank in this stacking sequence is the safest. The maximum error of the partial homogenization model is 6.2%, and the minimum error is 0.8%. The stress of stacking sequence a and stacking sequence b is large because the alternating hoop–helical winding has a gradient change in the thickness direction, and the fibers of the helical winding layers are not fully utilized, as shown in Figure 18.

Homogenization theory does not need to consider the distribution characteristics of materials but focuses on the macroscopic integrity. The homogenization model can not only predict the maximum stress along the fiber well but also effectively reduce the modeling complexity. As the number of composite layers becomes larger, the improvement becomes more obvious.

**Figure 19:**
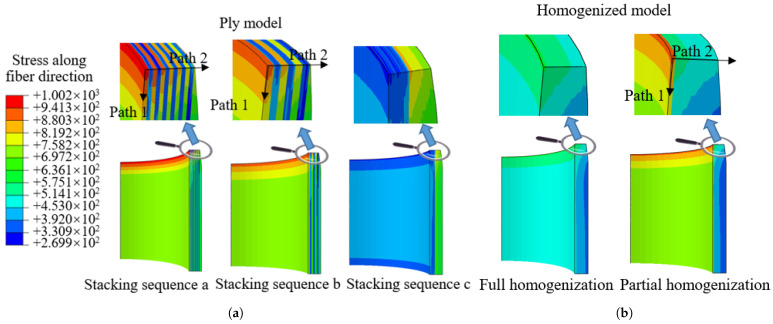
The stress along the fiber direction of the cylinder: (**a**) stacking sequences a, b, and c; (**b**) results of homogenization—full and partial—for stacking sequences a, b, and c.

**Figure 20:**
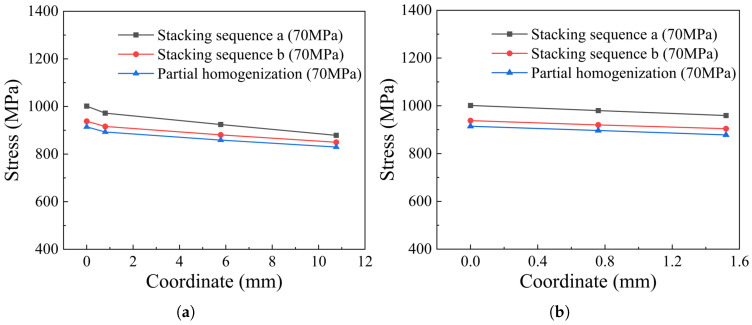
Comparison of stresses along the fiber direction. (**a**) Stress along the fiber direction of Path 1, (**b**) stress along the fiber direction of Path 2.

The distribution of stress along the fiber direction of cylinder thickness is shown in Figure 19. For the ply models, the stress along the fiber direction of the hoop winding layers is obviously larger than that of the helical winding layers, and the stress decreases with the increase in thickness. Partial homogenization can predict the burst pressure of the tank by the innermost layer, but full homogenization cannot reflect this characteristic. Localization of stress is an important aspect for understanding the distribution and variation trend. The max principal stresses of the original models on Path 1 and Path 2 (as shown in Figure 19) are compared with those of the partial homogenized model in Figure 20. Numerically, the max principal stress of the partially homogenized model is slightly smaller, so the margin needs to be introduced as a reference, and 1.044~1.084 is recommended. The maximum deviation is 8.7% in Figure 20. Meanwhile, it is found that the variation trend of stress along the fiber direction is consistent, which indicates that partial homogenization does not affect the elastic response or distribution trend of the innermost layer composite structure. When the burst pressure (185 MPa) is implemented, the maximum deviation between the ply model and the partially homogenized model decreases to 6.2%. It is shown that the partial homogenization method can predict the burst pressure under specific stacking sequences by the maximum stress criterion.

**Figure 21:**
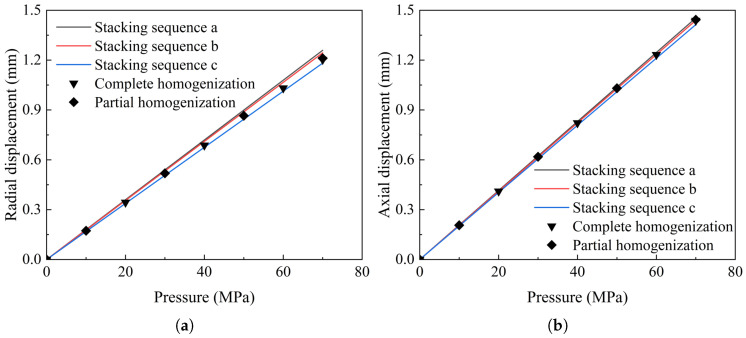
Comparison of radial displacement and axial displacement. (**a**) Radial displacement varies with internal pressure; (**b**) axial displacement varies with internal pressure.

The homogenization method can effectively characterize the overall properties of the composite layers, but the mechanical response of each layer cannot be characterized. For three different stacking sequences, namely, stacking sequence a, stacking sequence b and stacking sequence c, the radial displacement and axial displacement distributions of the tank at the internal pressure of 70 MPa are shown in Figure 21, and the displacement response of the homogenized model as a whole is accurate. Considering the stress distribution comprehensively, in theory, partial homogenization is more efficient than full homogenization.

### 5.2. Comparison of Burst Pressure

Considering that the homogenization effects of stacking sequence a, stacking sequence b, and stacking sequence c are better, a series of mechanical analyses are carried out under these three stacking sequences. The analysis of burst pressure cannot ignore the distribution of stress, and partial homogenization can retain the maximum stress region, but full homogenization cannot, which reflects the advantages of the partial homogenization method. The maximum stress criterion, as a common failure criterion, can be used to predict the failure of the composite layer of a pressure vessel. It is generally considered that the innermost layer requires special attention for composite part modeling. In this study, the two innermost layers of the helical or hoop winding are examined for failure evaluation. In the composite pressure vessel, the CFRP Hashin failure criterion is dominated by fiber tension:

(23) $$\mathrm{CRFT}={\left(\frac{\sigma_{1}}{X_{t}}\right)}^{2}>1$$

where $\sigma_{1}$ is the fiber-direction stress, and $X_{t}$ is the CFRP tensile strength, set as 2600 MPa [32].

**Figure 22:**
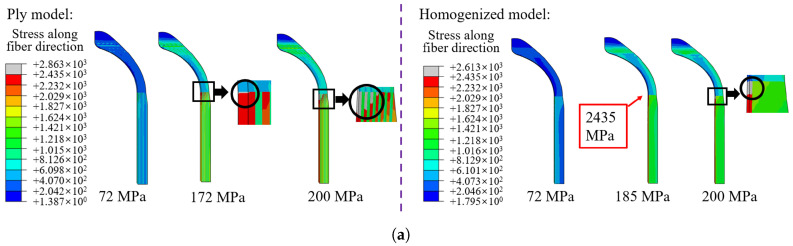

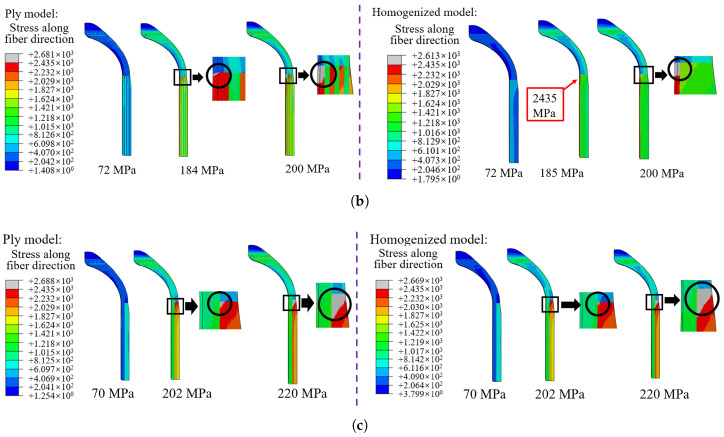
Comparison of damage in different modes. (**a**) Stacking sequence a, (**b**) stacking sequence b, (**c**) stacking sequence c.

The stacking sequence exerts a significant effect on the burst pressure. Numerical results of typical lay-up sequences a, b, and c indicate 172 MPa, 184 MPa, and 202 MPa burst pressure. This trend suggests that fewer alternating layers of hoop and helical winding result in higher burst pressure, which agrees well with the literature [16]. In order to analyze the burst pressure of the homogenized models, the maximum tensile stress criterion is used in this work. Furthermore, the validation of the homogenization method for predicting burst pressure under three different stacking sequences is discussed. The pressure on the inner surface of the liner is continuously increased to observe the minimum pressure that causes damage and damage expansion. Generally speaking, the internal pressure will increase in a small range from the appearance of damage to the rupture of the tank, but in practice, it is believed that the appearance of damage means the failure of the bearing performance of the tank. Therefore, the internal pressure at the time of damage can be regarded as the burst pressure. As shown in Figure 22, when the stress of the composite layer along the fiber direction is greater than the ultimate strength of the fiber, the deviation after homogenization of stacking sequence a is the largest, which is 7.56%. The burst pressure of the homogenization model of stacking sequence c is very close to that of the ply model. When the internal pressure increases, the damage expands along the thickness direction, and the hoop layers are destroyed before the helical layers. This result is consistent with the distribution of the maximum stress, indicating that the smaller the number of alternating layers of winding, the more accurate the homogenized model in predicting burst pressure.

### 5.3. Comparison of Liner Fatigue Life

Fatigue resistance is an important point that must be considered in the design of pressure vessels. Fatigue damage is difficult to detect, and the resulting damage is serious. Although the experimental results in Ref. [48] show that the 70 MPa hydrogen storage pressure vessel is constantly pressurized until fatigue failure, no obvious damage is found in the composite layer, and hydrogen will overflow in the form of foam. It is true that there are voids and defects in composite pressure vessels, which will initiate failure, as well as the interface, which is frequently the weakest link [49]. This section tries to focus on the stacking sequence effect on the liner fatigue life.

**Figure 23:**
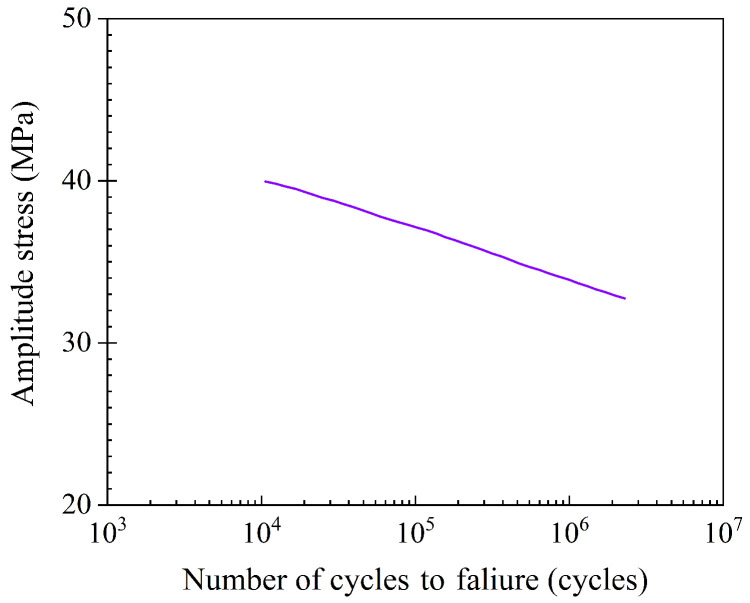
S-N curve of polyamide PA66 with zero stress ratio [50].

To verify that the prediction of the fatigue life of the homogenized tank is reliable, the commercial software ABAQUS^®^ and FE-SAFE are used for fatigue analysis. The liner is made of polyamide PA66, and the S-N curve obtained from a typical uniaxial test [50] is shown in Figure 23. The stress ratio is 0, so the Goodman algorithm [51] in FE-SAFE is introduced to correct the S-N curve.

**Figure 24:**
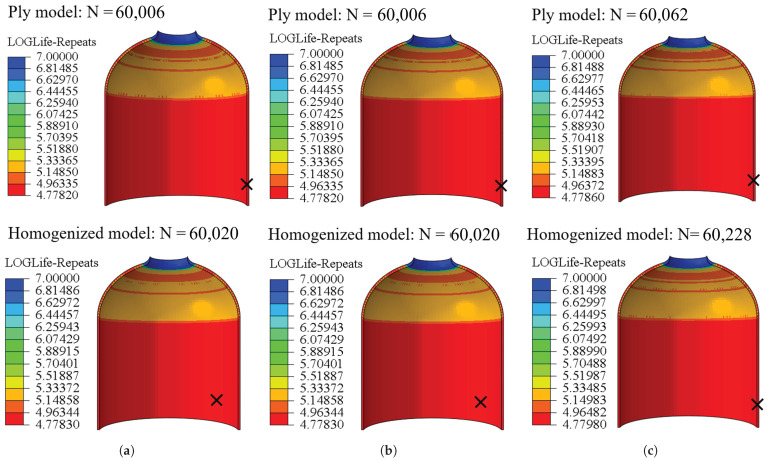
Comparison of fatigue life of three different stacking sequences, where N represents maximum number of loading cycles. (**a**) Stacking sequence a, (**b**) stacking sequence b, (**c**) stacking sequence c.

The stress distribution results from FE simulation and the mechanical properties of PA66 are input into FE-SAFE for fatigue life prediction. The predicted results from FE-SAFE are then imported into ABAQUS^®^ for post-processing visualization. Figure 24 shows the fatigue life of the model with three different stacking sequences, including the ply model and the homogenized model. For the ply model, different stacking sequences have little effect on the liner fatigue life, and the maximum deviation is only 0.1%. With a constant winding angle and different alternating layers, the fatigue life of the liners is the same (e.g., stacking sequences a and b). For the homogenized composite layers of the tank, the fatigue life of the liner is affected very little, and the maximum error occurs in stacking sequence c and is only 0.28%. The fatigue life nephogram distribution of the homogenized model is similar to that of the ply model, and fatigue failure of the whole cylinder occurs first.

## 6. Conclusions

In this study, a two-stage homogenization method, including in-plane RVE and inter-layer homogenization, is applied to cross-scale modeling of the CFRP stacking sequence in FW composite pressure vessels. A Type IV hydrogen tank is designed based on netting theory, where the traditional ply model of the CFRP layer is validated in terms of strain–pressure response. In-plane and inter-layer homogenization is further conducted to explore the CFRP lay-up sequence’s effect on the composite pressure vessel’s mechanical response, burst pressure, and fatigue life. Conclusions are drawn as follows:

(1) In the frame of the homogenization approach, the laminate can represent the in-plane FW cross-undulation RVE structure. Therefore, the effective elastic modulus of the CFRP layer can be modeled as the modulus of a laminate layer in composite pressure vessel simulations.
(2) Inter-layer homogenization can model the structural response, including the radial and axial displacements, consistent with the validated ply model. Keeping the two innermost layers as mesoscale plies, partial homogenization for the alternate CFRP layers can predict the burst pressure with tensile strength of the Hashin failure criterion with a deviation of less than 7.56%. The model with fewer alternating hoop and helical winding layers has higher burst pressure, which needs further experimental validation.
(3) By coupling ABAQUS^®^ with FE-SAFE and incorporating an S-N curve for polyamide PA66, fatigue life analysis of the Type IV hydrogen composite tank liner is conducted. The results indicate that the fatigue cycles of the liner exhibit only 0.28% variation across different stacking sequences, demonstrating that homogenization has a negligible impact on lifecycle predictions.

## Figures and Tables

**Table 1:** Material properties.

Material	Density $\left(g/{\mathrm{cm}}^{3}\right)$	*E* (MPa)	ν
T700SC-12K	1.8	230,000	0.3
914 epoxy	1.14	4000	0.39

**Table 2:** Homogenized material properties at the microscale.

$E_{1}\;\left(\mathrm{MPa}\right)$	$E_{2}\;\left(\mathrm{MPa}\right)$	$E_{3}\;\left(\mathrm{MPa}\right)$	$\nu_{12}$	$\nu_{13}$	$\nu_{23}$	$G_{12}\;\left(\mathrm{MPa}\right)$	$G_{13}\;\left(\mathrm{MPa}\right)$	$G_{23}\;\left(\mathrm{MPa}\right)$
115,943	16,077	16,077	0.34	0.34	0.39	4201	4201	3124

**Table 3:** Basic parameters of the tank.

$P_{\max}\;\left(\mathrm{MPa}\right)$	$r_{0}\;\left(\mathrm{mm}\right)$	R(mm)	b(mm)	$t_{\theta }\;\left(\mathrm{mm}\right)$	$t_{\alpha }\;\left(\mathrm{mm}\right)$	$r_{b}\;\left(\mathrm{mm}\right)$	$r_{2b}\;\left(\mathrm{mm}\right)$	α	$t_{P}\;\left(\mathrm{mm}\right)$
185	35	134	6.4	12.3	8.6	41.4	47.8	15.1°	0.76

**Table 4:** The effective material parameters of the homogenization of different RVEs.

	$E_{1}\;\left(\mathrm{MPa}\right)$	$E_{2}\;\left(\mathrm{MPa}\right)$	$E_{3}\;\left(\mathrm{MPa}\right)$	$\nu_{12}$	$\nu_{13}$	$\nu_{23}$	$G_{12}\;\left(\mathrm{MPa}\right)$	$G_{13}\;\left(\mathrm{MPa}\right)$	$G_{23}\;\left(\mathrm{MPa}\right)$
${\mathrm{RVE}}_{a}$	25,569	25,569	8257	0.10	0.52	0.52	2089	1901	1901
${\mathrm{RVE}}_{b}$	24,478	24,470	8153	0.11	0.53	0.53	2085	1916	1916

**Table 5:** The variation in the effective modulus at the dome with the winding angle.

No.	Angle	$E_{11}\;\left(\mathrm{GPa}\right)$	$E_{22}\;\left(\mathrm{GPa}\right)$	$E_{33}\;\left(\mathrm{GPa}\right)$	$G_{12}\;\left(\mathrm{GPa}\right)$	$G_{13}\;\left(\mathrm{GPa}\right)$	$G_{23}\;\left(\mathrm{GPa}\right)$	Graphic
1#	90°	16.1	16.1	116	3.12	4.20	4.20	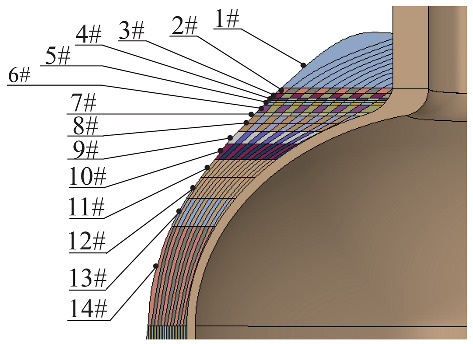
2#	76.5°	15.3	16.2	99.4	3.17	9.70	4.12	
3#	71.1°	14.5	16.3	83.0	3.21	14.2	4.05	
4#	67.1°	13.9	16.5	68.4	3.25	17.9	3.99	
5#	63.8°	13.4	16.6	56.0	3.29	20.9	3.94	
6#	60°	12.8	16.9	42.7	3.34	24.1	3.87	
7#	51.1°	12.7	17.5	21.5	3.48	29.6	3.70	
8#	44.4°	15.5	17.8	14.7	3.56	30.8	3.57	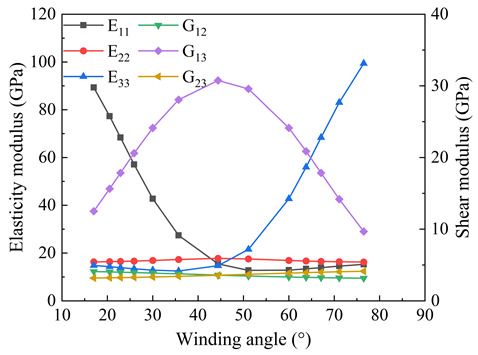
9#	35.7°	27.4	17.3	12.5	3.76	28.1	3.42	
10#	30°	42.7	16.9	12.8	3.87	24.1	3.34	
11#	25.9°	57.1	16.6	13.4	3.94	20.6	3.29	
12#	22.9°	68.4	16.5	13.9	3.09	17.9	3.25	
13#	20.5°	77.3	16.4	14.3	4.03	15.6	3.23	
14#	17°	89.3	16.3	14.8	4.08	12.5	3.19	

## Data Availability

The original contributions presented in this study are included in the article. Further inquiries can be directed to the corresponding authors.
